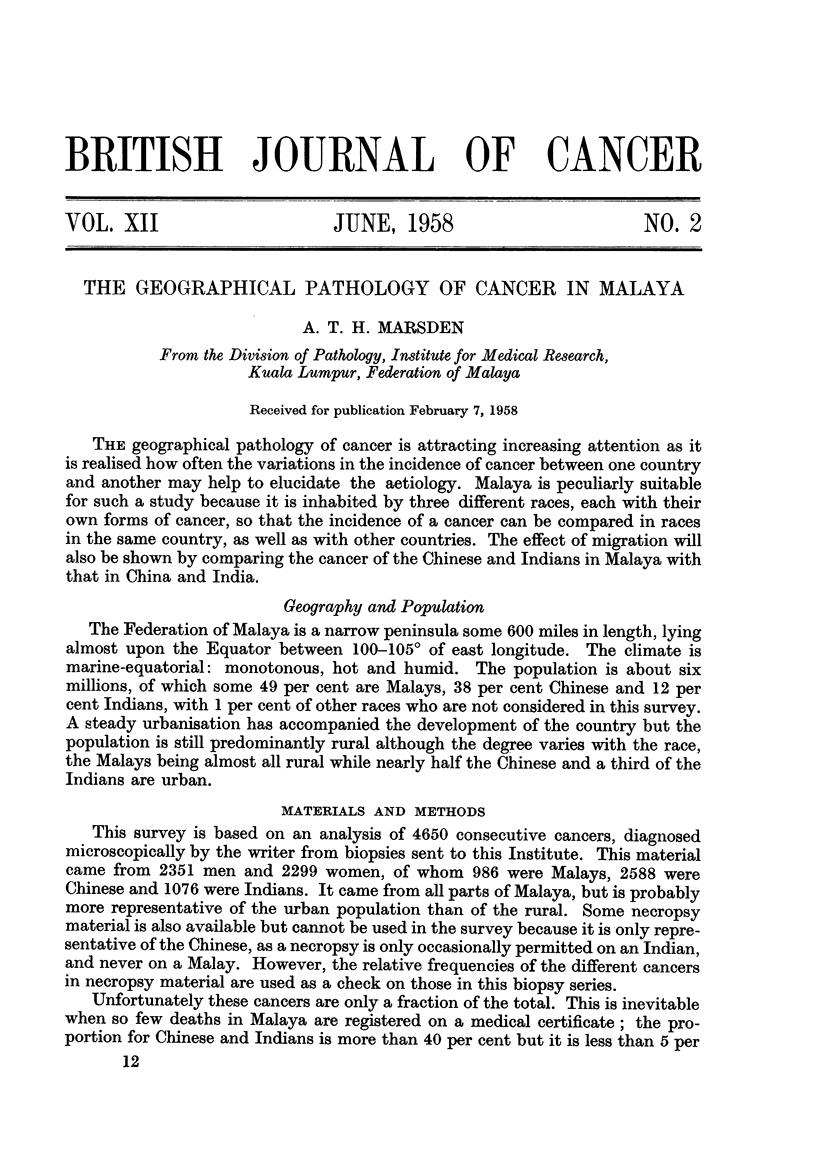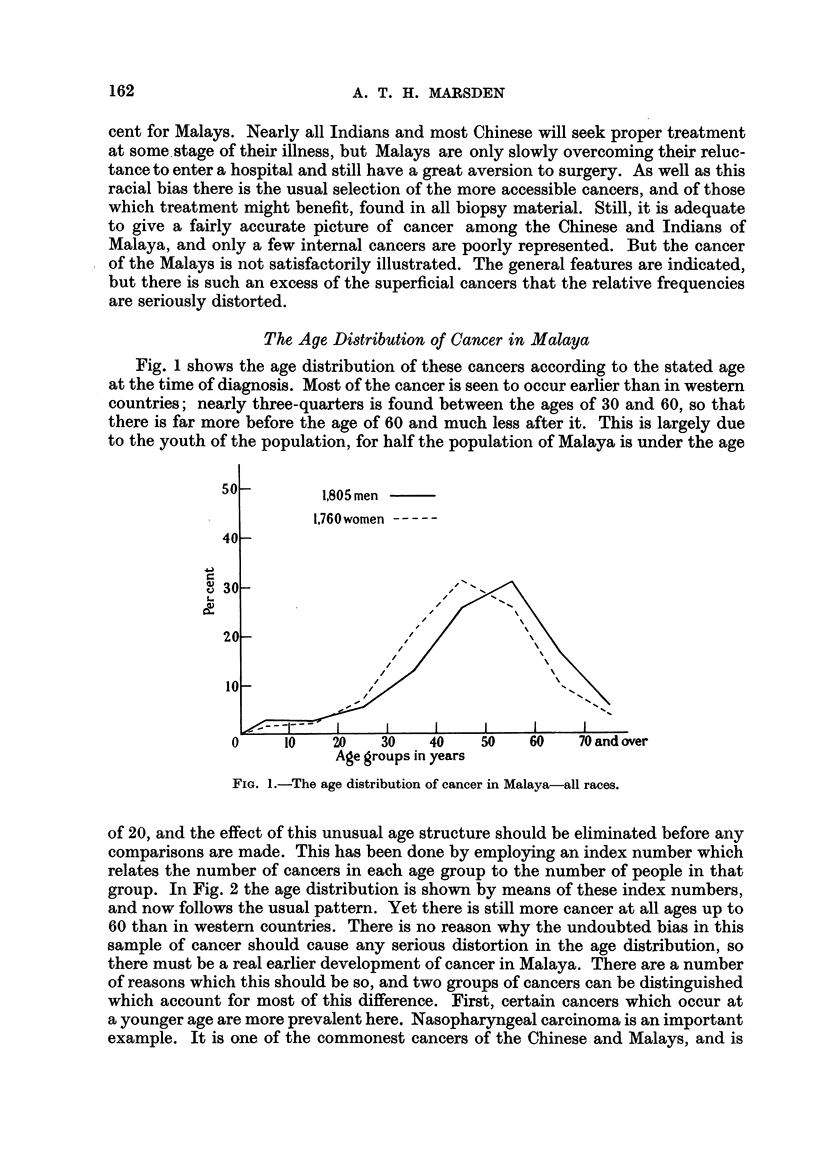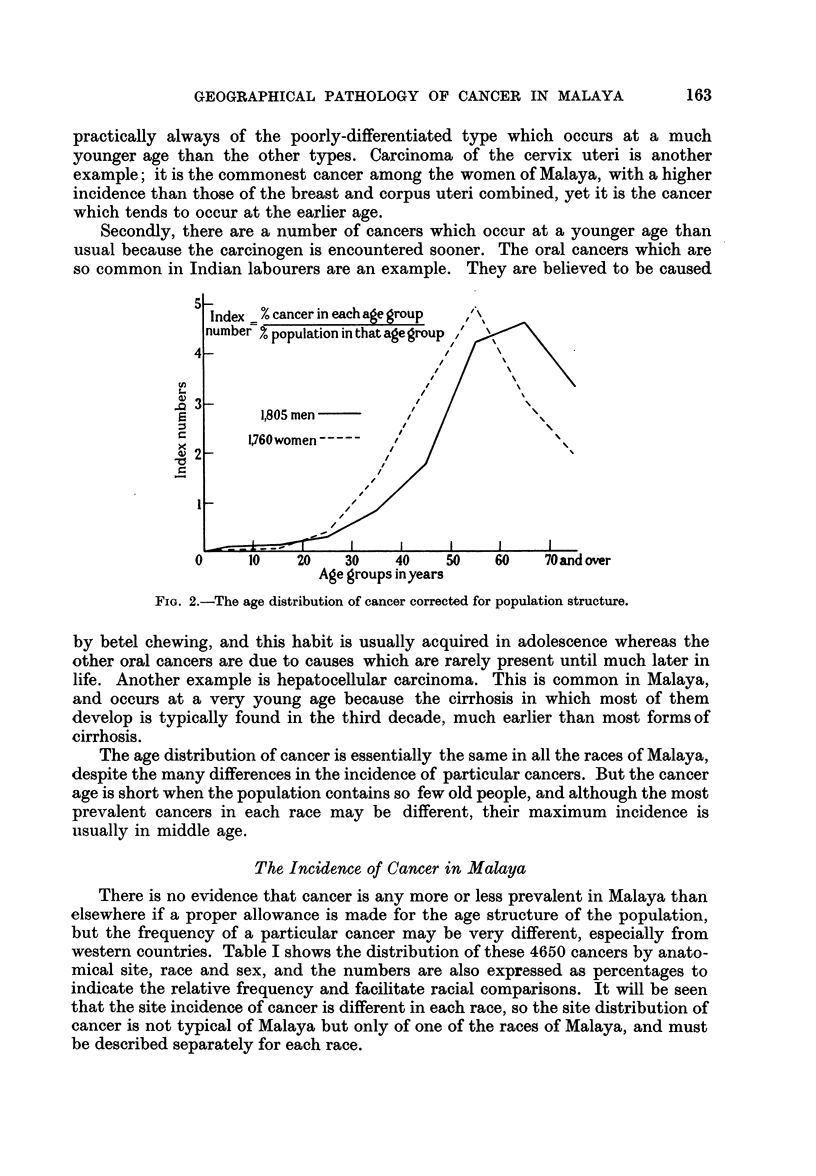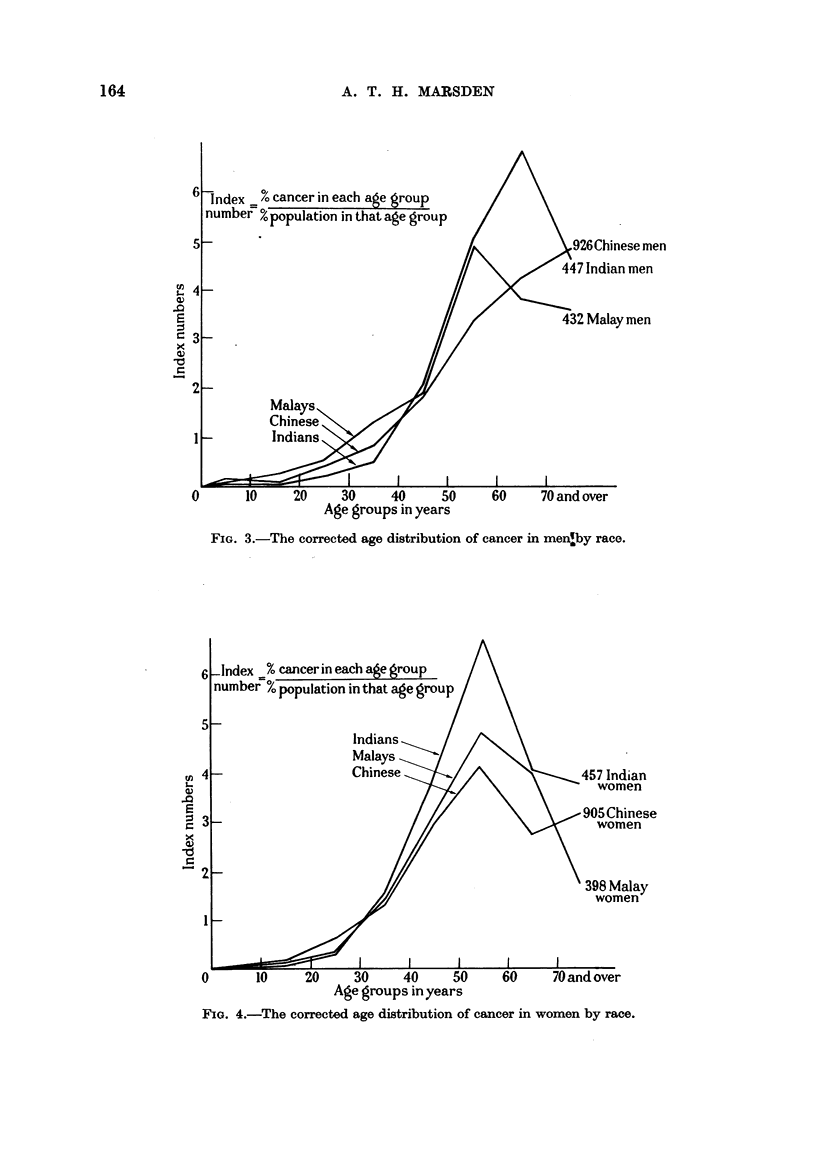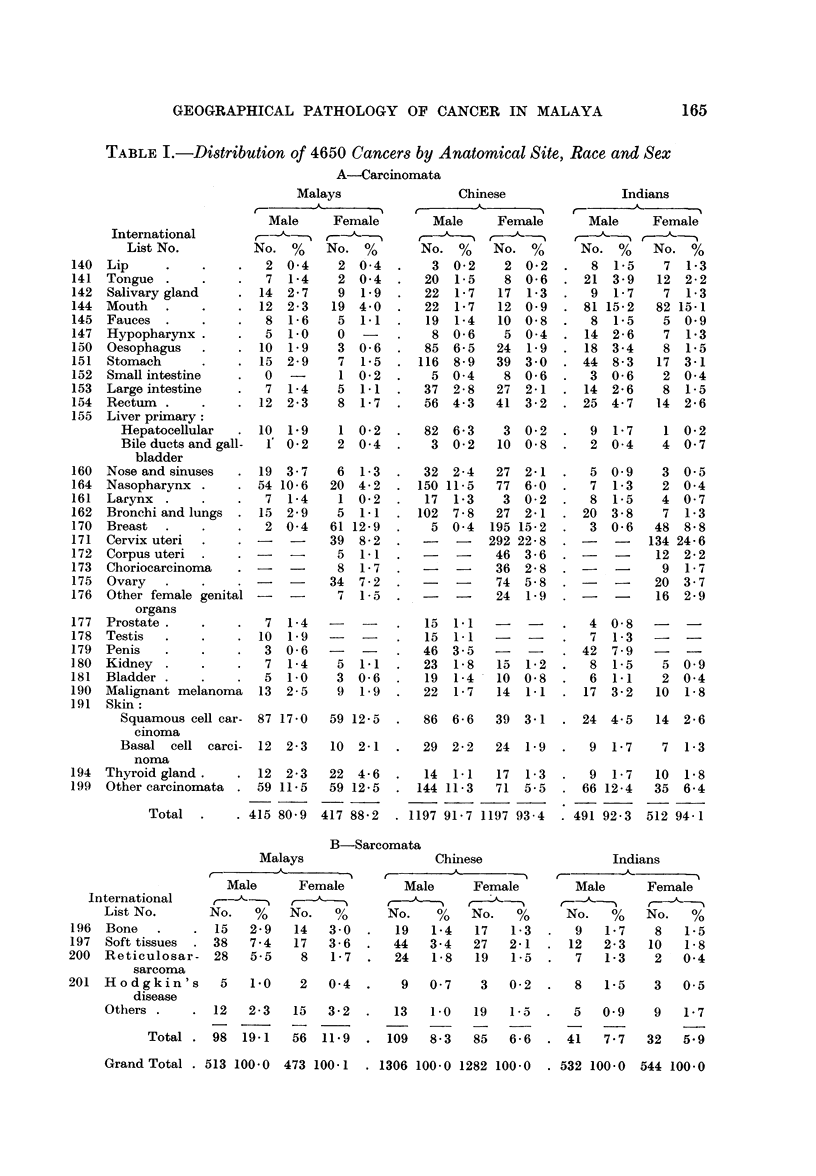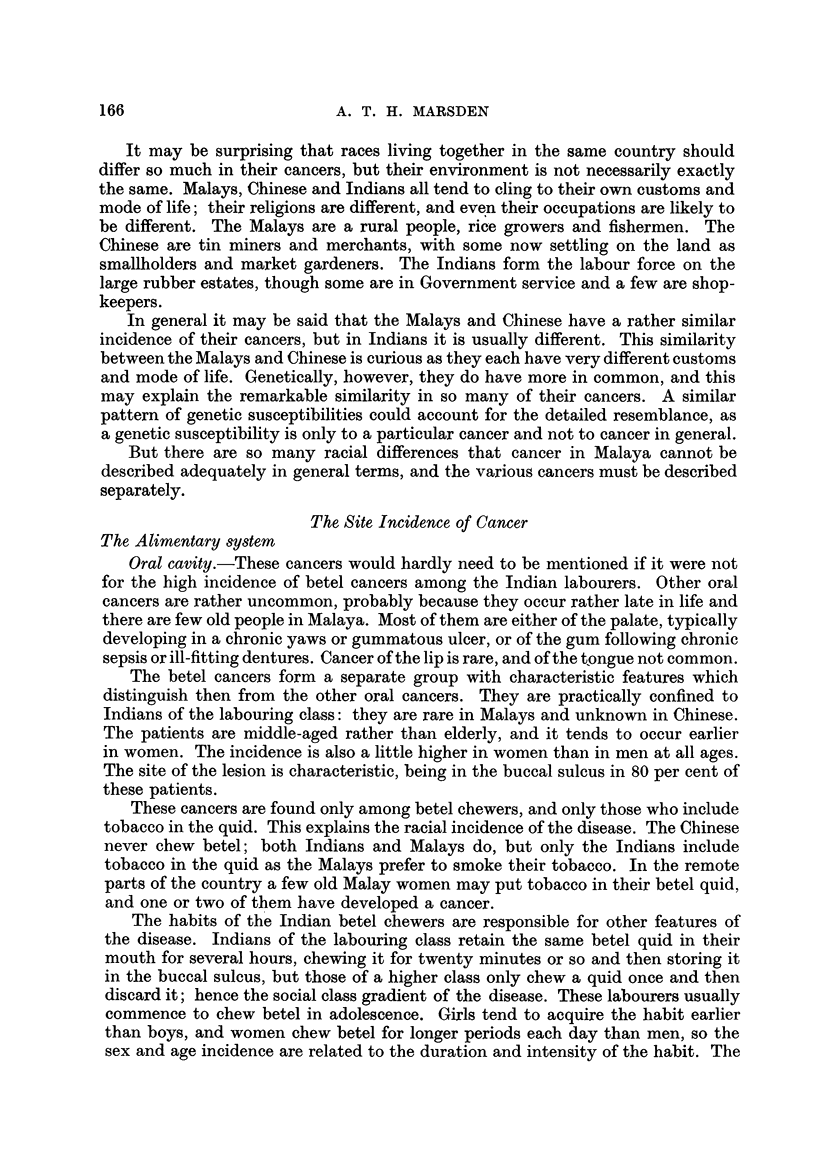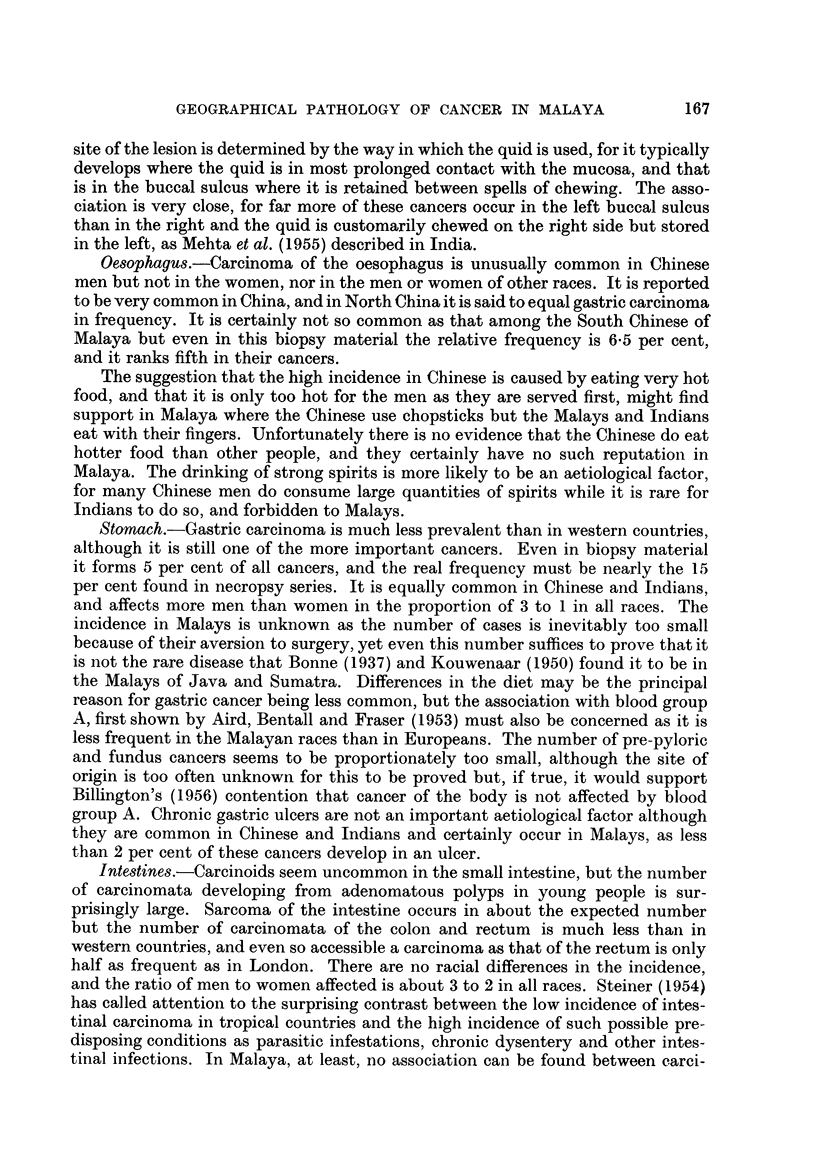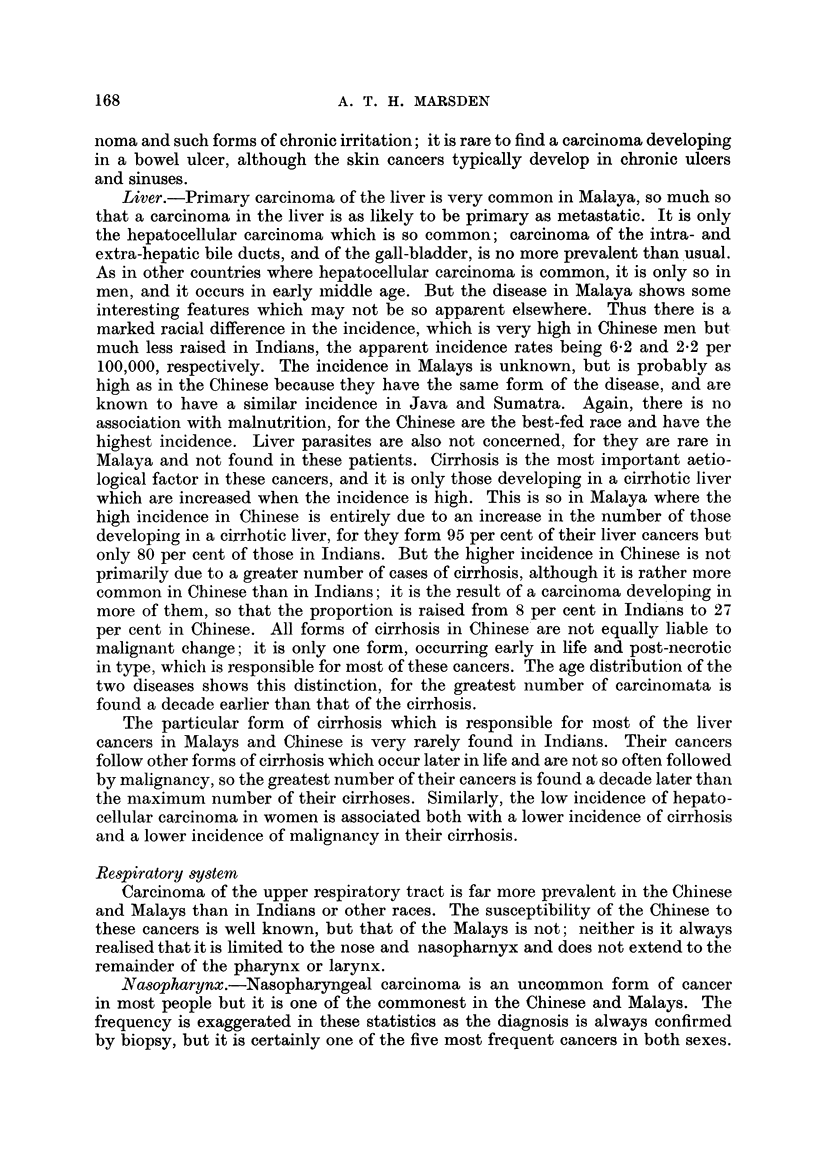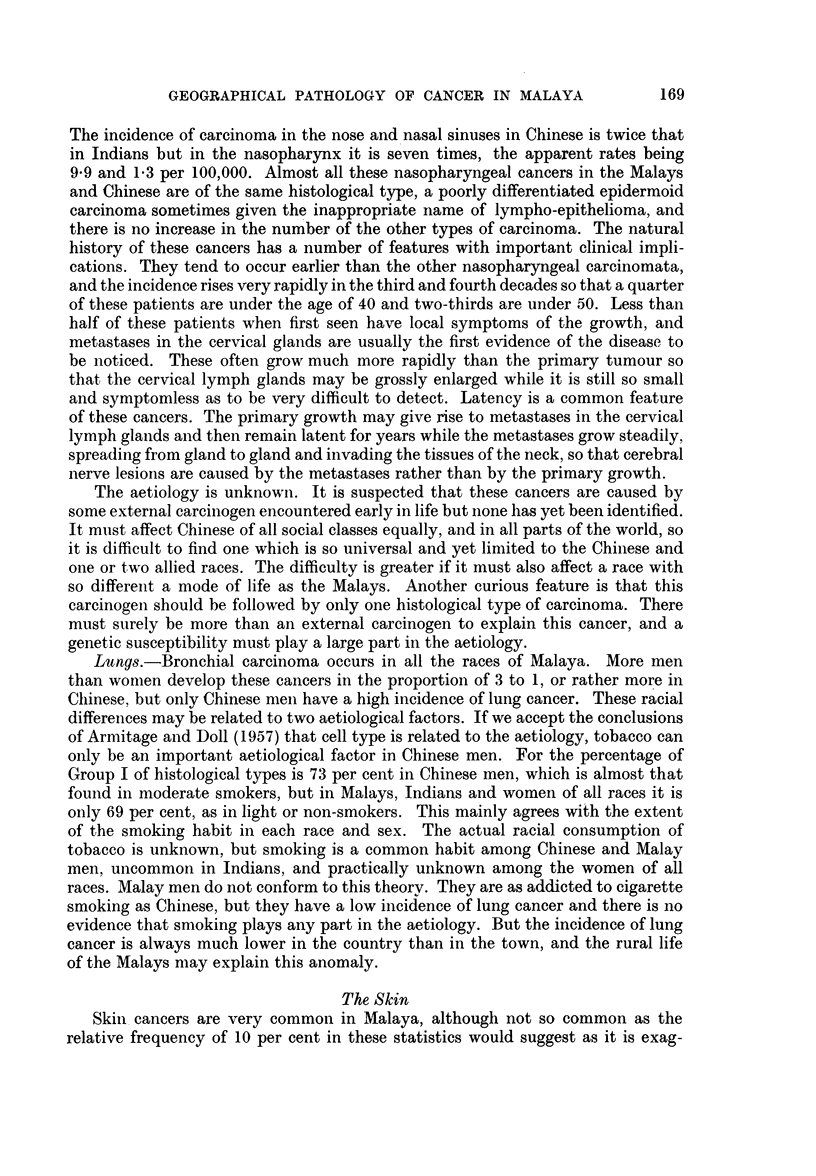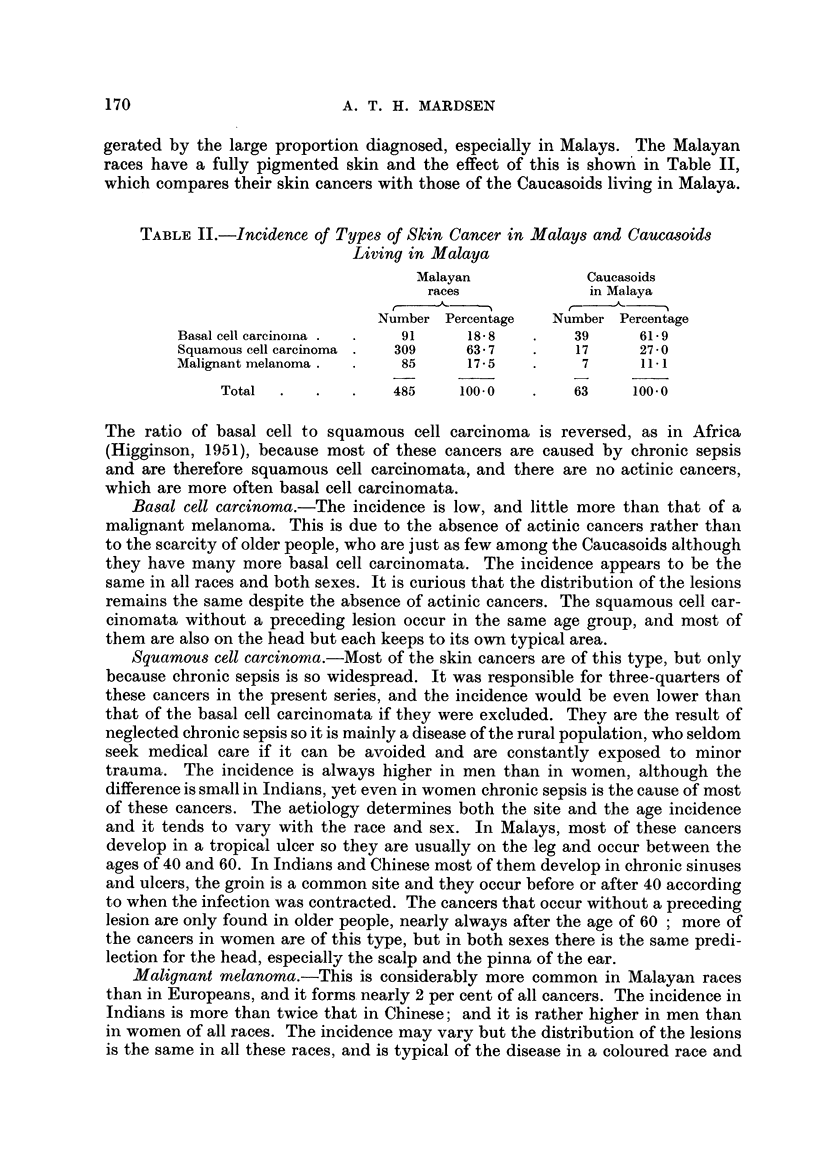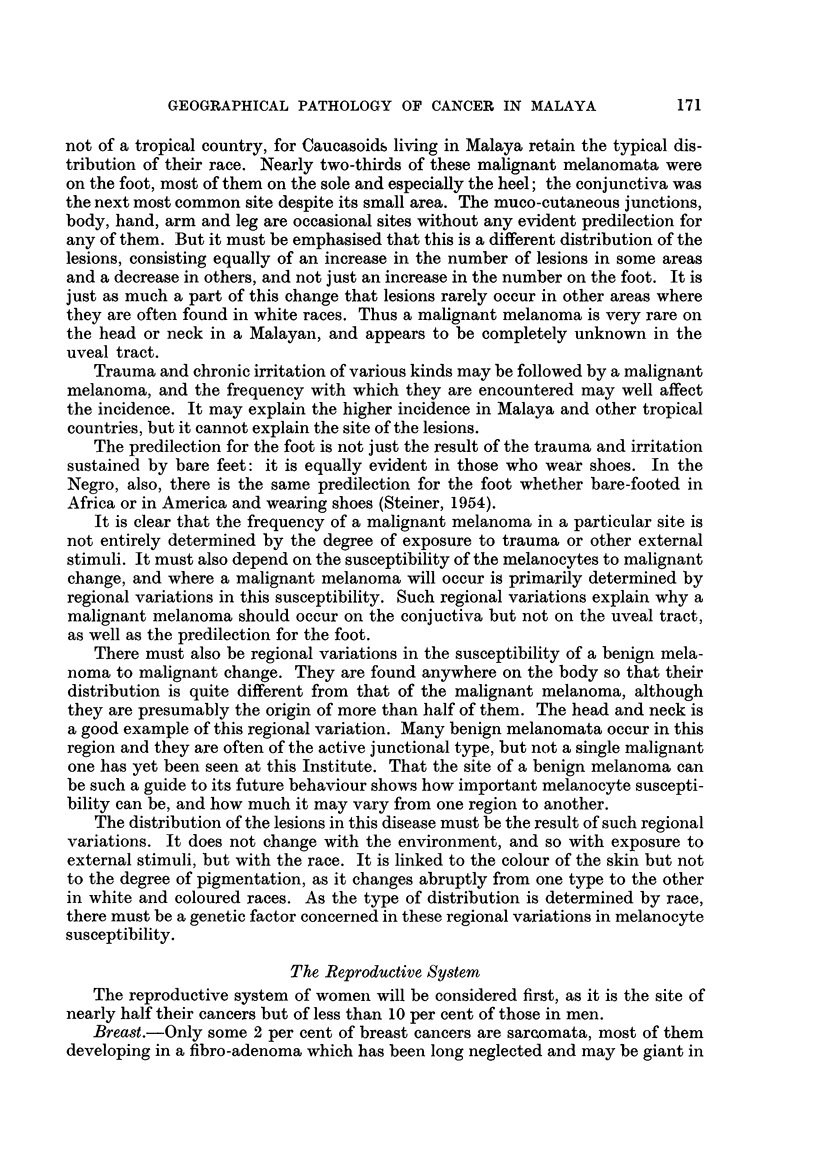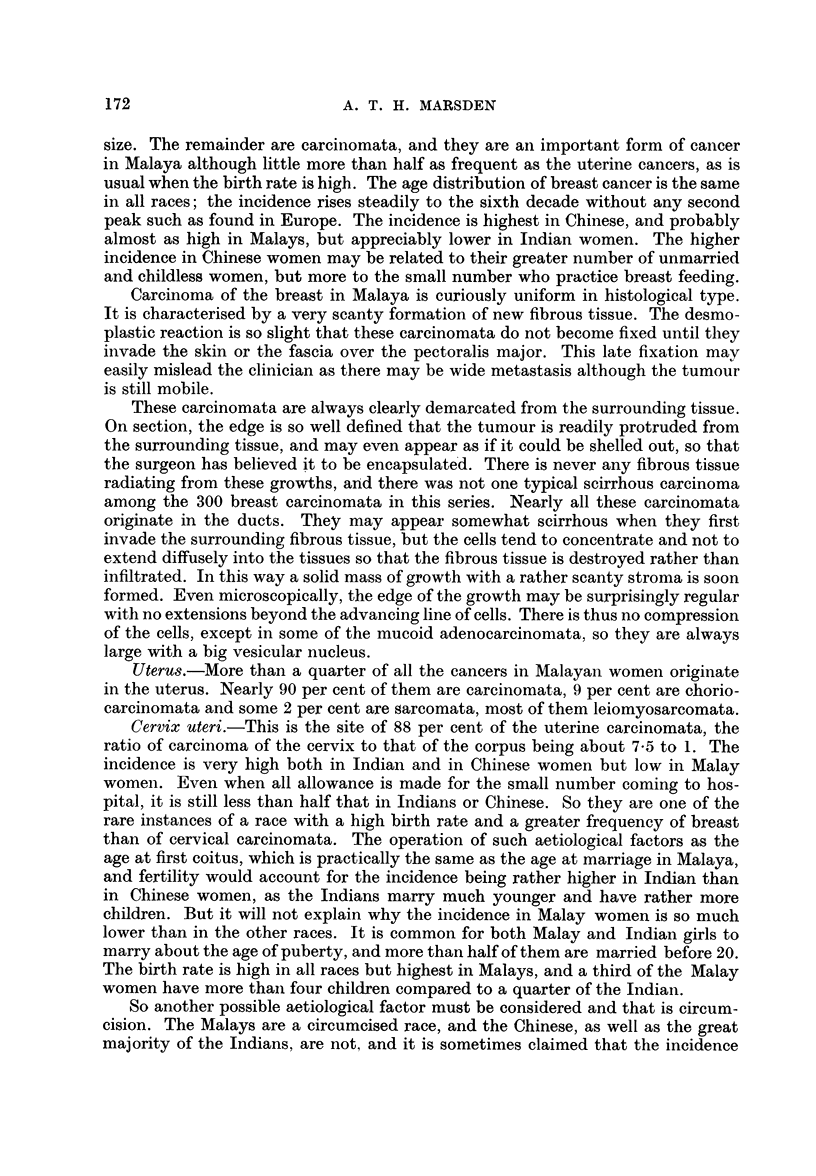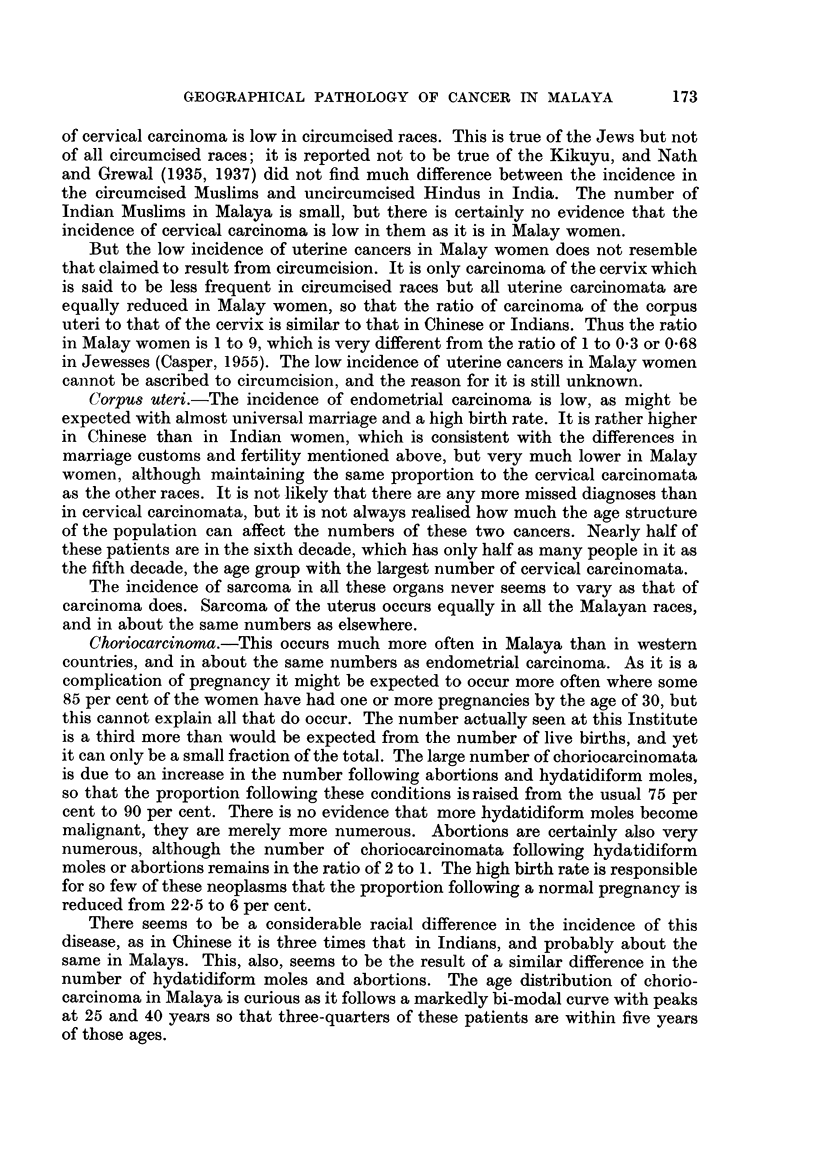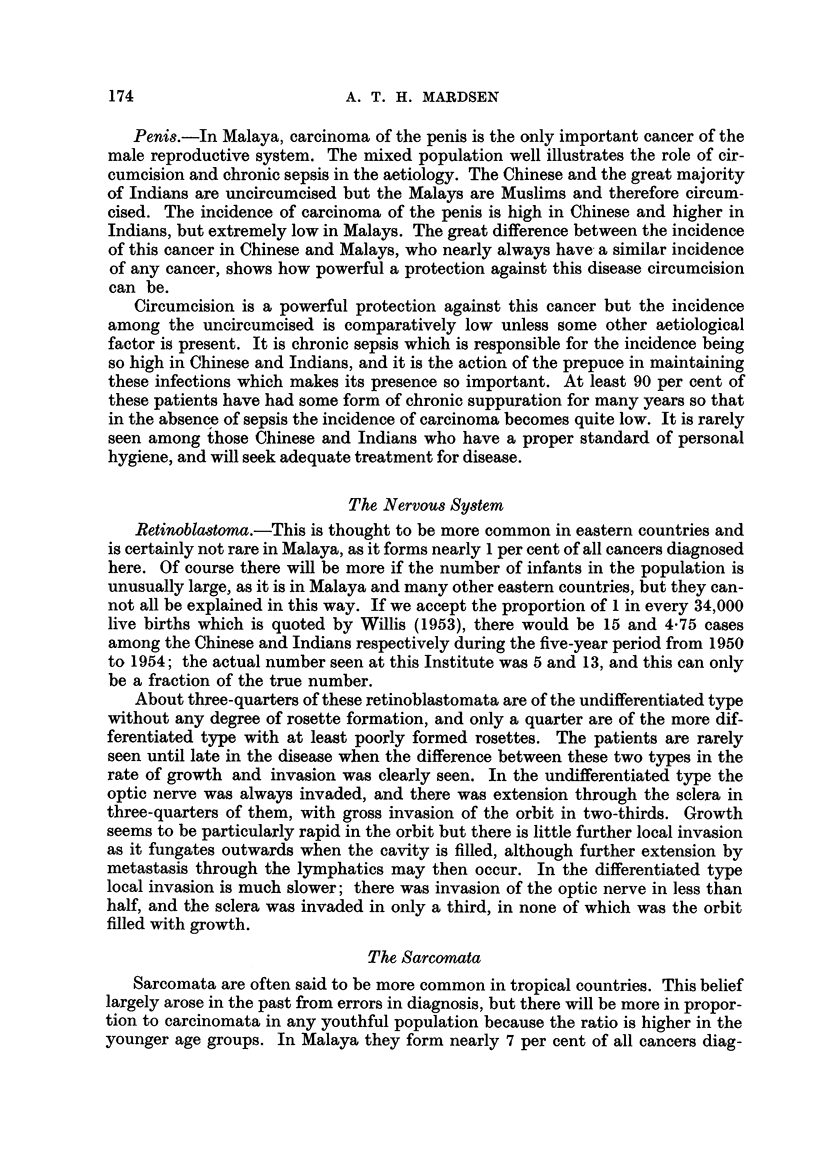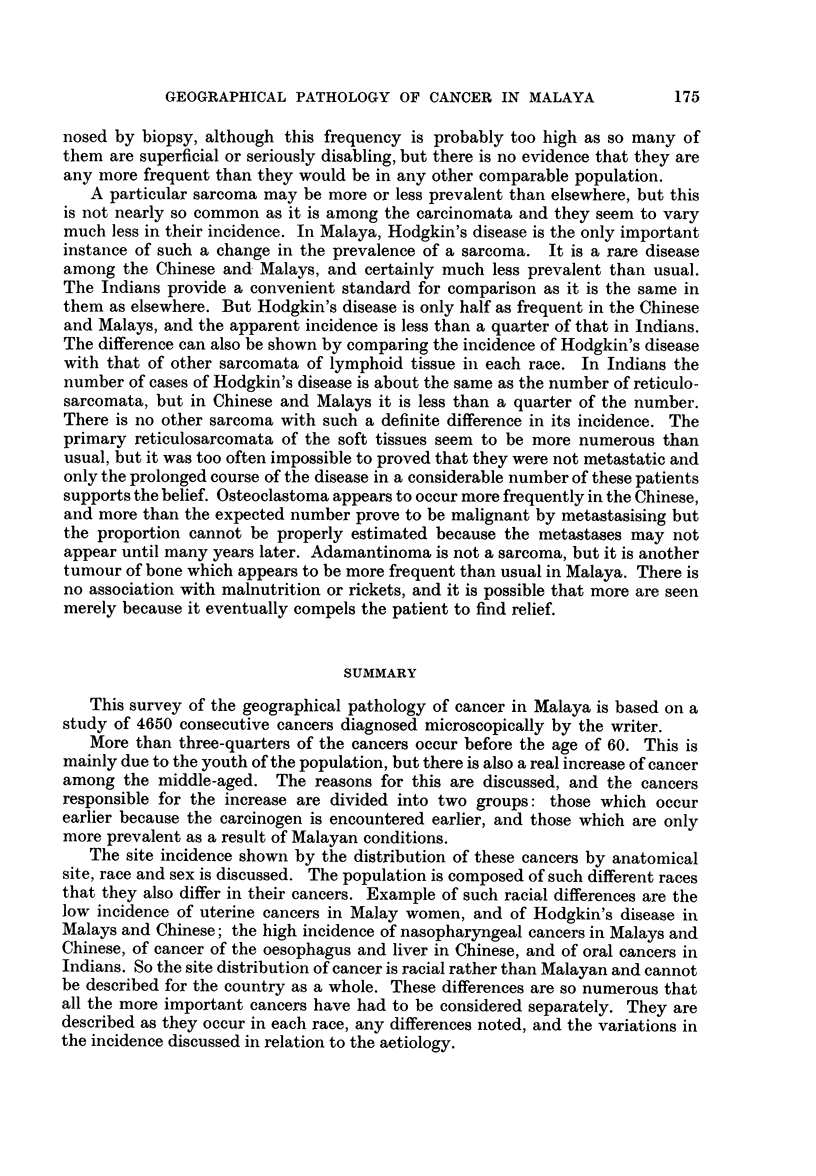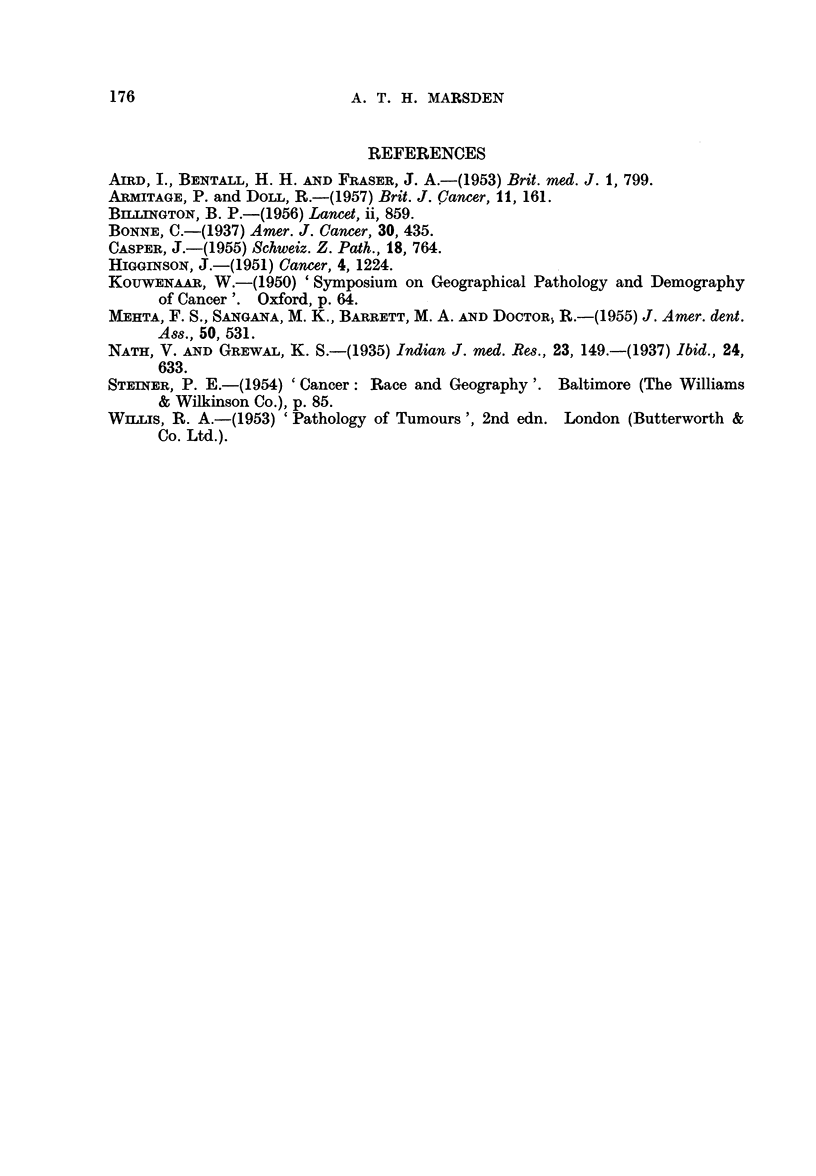# The Geographical Pathology of Cancer in Malaya

**DOI:** 10.1038/bjc.1958.20

**Published:** 1958-06

**Authors:** A. T. H. Marsden


③ BRITISH JOURNAL OF CANCER

VOL. XII JUNE, 1958 NO. 2

### THE GEOGRAPHICAL PATHOLOGY OF CANCER IN MALAYA

A. T. H. MARSDEN

*From the Division of Pathology, Institute for Medical Research, Kuala Lumpur, Federation of Malaya*

Received for publication February 7, 1958

THE geographical pathology of cancer is attracting increasing attention as it is realised how often the variations in the incidence of cancer between one country and another may help to elucidate the aetiology. Malaya is peculiarly suitable for such a study because it is inhabited by three different races, each with their own forms of cancer, so that the incidence of a cancer can be compared in races in the same country, as well as with other countries. The effect of migration will also be shown by comparing the cancer of the Chinese and Indians in Malaya with that in China and India.

### *Geography and Population*

The Federation of Malaya is a narrow peninsula some 600 miles in length, lying almost upon the Equator between 100–105° of east longitude. The climate is marine-equatorial: monotonous, hot and humid. The population is about six millions, of which some 49 per cent are Malays, 38 per cent Chinese and 12 per cent Indians, with 1 per cent of other races who are not considered in this survey. A steady urbanisation has accompanied the development of the country but the population is still predominantly rural although the degree varies with the race, the Malays being almost all rural while nearly half the Chinese and a third of the Indians are urban.

### MATERIALS AND METHODS

This survey is based on an analysis of 4650 consecutive cancers, diagnosed microscopically by the writer from biopsies sent to this Institute. This material came from 2351 men and 2299 women, of whom 986 were Malays, 2588 were Chinese and 1076 were Indians. It came from all parts of Malaya, but is probably more representative of the urban population than of the rural. Some necropsy material is also available but cannot be used in the survey because it is only representative of the Chinese, as a necropsy is only occasionally permitted on an Indian, and never on a Malay. However, the relative frequencies of the different cancers in necropsy material are used as a check on those in this biopsy series.

Unfortunately these cancers are only a fraction of the total. This is inevitable when so few deaths in Malaya are registered on a medical certificate ; the proportion for Chinese and Indians is more than 40 per cent but it is less than 5 per cent for Malays. Nearly all Indians and most Chinese will seek proper treatment at some stage of their illness, but Malays are only slowly overcoming their reluctance to enter a hospital and still have a great aversion to surgery. As well as this racial bias there is the usual selection of the more accessible cancers, and of those which treatment might benefit, found in all biopsy material. Still, it is adequate to give a fairly accurate picture of cancer among the Chinese and Indians of Malaya, and only a few internal cancers are poorly represented. But the cancer of the Malays is not satisfactorily illustrated. The general features are indicated, but there is such an excess of the superficial cancers that the relative frequencies are seriously distorted.

*The Age Distribution of Cancer in Malaya*

Fig. 1 shows the age distribution of these cancers according to the stated age at the time of diagnosis. Most of the cancer is seen to occur earlier than in western countries; nearly three-quarters is found between the ages of 30 and 60, so that there is far more before the age of 60 and much less after it. This is largely due to the youth of the population, for half the population of Malaya is under the age

FIG. 1.—The age distribution of cancer in Malaya—all races.

of 20, and the effect of this unusual age structure should be eliminated before any comparisons are made. This has been done by employing an index number which relates the number of cancers in each age group to the number of people in that group. In Fig. 2 the age distribution is shown by means of these index numbers, and now follows the usual pattern. Yet there is still more cancer at all ages up to 60 than in western countries. There is no reason why the undoubted bias in this sample of cancer should cause any serious distortion in the age distribution, so there must be a real earlier development of cancer in Malaya. There are a number of reasons which this should be so, and two groups of cancers can be distinguished which account for most of this difference. First, certain cancers which occur at a younger age are more prevalent here. Nasopharyngeal carcinoma is an important example. It is one of the commonest cancers of the Chinese and Malays, and is practically always of the poorly-differentiated type which occurs at a much younger age than the other types. Carcinoma of the cervix uteri is another example; it is the commonest cancer among the women of Malaya, with a higher incidence than those of the breast and corpus uteri combined, yet it is the cancer which tends to occur at the earlier age.

Secondly, there are a number of cancers which occur at a younger age than usual because the carcinogen is encountered sooner. The oral cancers which are so common in Indian labourers are an example. They are believed to be caused

FIG. 2.—The age distribution of cancer corrected for population structure.

by betel chewing, and this habit is usually acquired in adolescence whereas the other oral cancers are due to causes which are rarely present until much later in life. Another example is hepatocellular carcinoma. This is common in Malaya, and occurs at a very young age because the cirrhosis in which most of them develop is typically found in the third decade, much earlier than most forms of cirrhosis.

The age distribution of cancer is essentially the same in all the races of Malaya, despite the many differences in the incidence of particular cancers. But the cancer age is short when the population contains so few old people, and although the most prevalent cancers in each race may be different, their maximum incidence is usually in middle age.

### *The Incidence of Cancer in Malaya*

There is no evidence that cancer is any more or less prevalent in Malaya than elsewhere if a proper allowance is made for the age structure of the population, but the frequency of a particular cancer may be very different, especially from western countries. Table I shows the distribution of these 4650 cancers by anatomical site, race and sex, and the numbers are also expressed as percentages to indicate the relative frequency and facilitate racial comparisons. It will be seen that the site incidence of cancer is different in each race, so the site distribution of cancer is not typical of Malaya but only of one of the races of Malaya, and must be described separately for each race.

FIG. 3.—The corrected age distribution of cancer in men by race.

FIG. 4.—The corrected age distribution of cancer in women by race.

TABLE I.—*Distribution of* 4650 *Cancers by Anatomical Site, Race and Sex*

A—Carcinomata

| International List No. | | Malays | | | | Chinese | | | | Indians | | | |
|---|---|---|---|---|---|---|---|---|---|---|---|---|---|
| | | Male | | Female | | Male | | Female | | Male | | Female | |
| | | No. | % | No. | % | No. | % | No. | % | No. | % | No. | % |
| 140 | Lip | 2 | 0·4 | 2 | 0·4 | 3 | 0·2 | 2 | 0·2 | 8 | 1·5 | 7 | 1·3 |
| 141 | Tongue | 7 | 1·4 | 2 | 0·4 | 20 | 1·5 | 8 | 0·6 | 21 | 3·9 | 12 | 2·2 |
| 142 | Salivary gland | 14 | 2·7 | 9 | 1·9 | 22 | 1·7 | 17 | 1·3 | 9 | 1·7 | 7 | 1·3 |
| 144 | Mouth | 12 | 2·3 | 19 | 4·0 | 22 | 1·7 | 12 | 0·9 | 81 | 15·2 | 82 | 15·1 |
| 145 | Fauces | 8 | 1·6 | 5 | 1·1 | 19 | 1·4 | 10 | 0·8 | 8 | 1·5 | 5 | 0·9 |
| 147 | Hypopharynx | 5 | 1·0 | 0 | — | 8 | 0·6 | 5 | 0·4 | 14 | 2·6 | 7 | 1·3 |
| 150 | Oesophagus | 10 | 1·9 | 3 | 0·6 | 85 | 6·5 | 24 | 1·9 | 18 | 3·4 | 8 | 1·5 |
| 151 | Stomach | 15 | 2·9 | 7 | 1·5 | 116 | 8·9 | 39 | 3·0 | 44 | 8·3 | 17 | 3·1 |
| 152 | Small intestine | 0 | — | 1 | 0·2 | 5 | 0·4 | 8 | 0·6 | 3 | 0·6 | 2 | 0·4 |
| 153 | Large intestine | 7 | 1·4 | 5 | 1·1 | 37 | 2·8 | 27 | 2·1 | 14 | 2·6 | 8 | 1·5 |
| 154 | Rectum | 12 | 2·3 | 8 | 1·7 | 56 | 4·3 | 41 | 3·2 | 25 | 4·7 | 14 | 2·6 |
| 155 | Liver primary : | | | | | | | | | | | | |
| | Hepatocellular | 10 | 1·9 | 1 | 0·2 | 82 | 6·3 | 3 | 0·2 | 9 | 1·7 | 1 | 0·2 |
| | Bile ducts and gall-bladder | 1 | 0·2 | 2 | 0·4 | 3 | 0·2 | 10 | 0·8 | 2 | 0·4 | 4 | 0·7 |
| 160 | Nose and sinuses | 19 | 3·7 | 6 | 1·3 | 32 | 2·4 | 27 | 2·1 | 5 | 0·9 | 3 | 0·5 |
| 164 | Nasopharynx | 54 | 10·6 | 20 | 4·2 | 150 | 11·5 | 77 | 6·0 | 7 | 1·3 | 2 | 0·4 |
| 161 | Larynx | 7 | 1·4 | 1 | 0·2 | 17 | 1·3 | 3 | 0·2 | 8 | 1·5 | 4 | 0·7 |
| 162 | Bronchi and lungs | 15 | 2·9 | 5 | 1·1 | 102 | 7·8 | 27 | 2·1 | 20 | 3·8 | 7 | 1·3 |
| 170 | Breast | 2 | 0·4 | 61 | 12·9 | 5 | 0·4 | 195 | 15·2 | 3 | 0·6 | 48 | 8·8 |
| 171 | Cervix uteri | — | — | 39 | 8·2 | — | — | 292 | 22·8 | — | — | 134 | 24·6 |
| 172 | Corpus uteri | — | — | 5 | 1·1 | — | — | 46 | 3·6 | — | — | 12 | 2·2 |
| 173 | Choriocarcinoma | — | — | 8 | 1·7 | — | — | 36 | 2·8 | — | — | 9 | 1·7 |
| 175 | Ovary | — | — | 34 | 7·2 | — | — | 74 | 5·8 | — | — | 20 | 3·7 |
| 176 | Other female genital organs | — | — | 7 | 1·5 | — | — | 24 | 1·9 | — | — | 16 | 2·9 |
| 177 | Prostate | 7 | 1·4 | — | — | 15 | 1·1 | — | — | 4 | 0·8 | — | — |
| 178 | Testis | 10 | 1·9 | — | — | 15 | 1·1 | — | — | 7 | 1·3 | — | — |
| 179 | Penis | 3 | 0·6 | — | — | 46 | 3·5 | — | — | 42 | 7·9 | — | — |
| 180 | Kidney | 7 | 1·4 | 5 | 1·1 | 23 | 1·8 | 15 | 1·2 | 8 | 1·5 | 5 | 0·9 |
| 181 | Bladder | 5 | 1·0 | 3 | 0·6 | 19 | 1·4 | 10 | 0·8 | 6 | 1·1 | 2 | 0·4 |
| 190 | Malignant melanoma | 13 | 2·5 | 9 | 1·9 | 22 | 1·7 | 14 | 1·1 | 17 | 3·2 | 10 | 1·8 |
| 191 | Skin : | | | | | | | | | | | | |
| | Squamous cell carcinoma | 87 | 17·0 | 59 | 12·5 | 86 | 6·6 | 39 | 3·1 | 24 | 4·5 | 14 | 2·6 |
| | Basal cell carcinoma | 12 | 2·3 | 10 | 2·1 | 29 | 2·2 | 24 | 1·9 | 9 | 1·7 | 7 | 1·3 |
| 194 | Thyroid gland | 12 | 2·3 | 22 | 4·6 | 14 | 1·1 | 17 | 1·3 | 9 | 1·7 | 10 | 1·8 |
| 199 | Other carcinomata | 59 | 11·5 | 59 | 12·5 | 144 | 11·3 | 71 | 5·5 | 66 | 12·4 | 35 | 6·4 |
| | Total | 415 | 80·9 | 417 | 88·2 | 1197 | 91·7 | 1197 | 93·4 | 491 | 92·3 | 512 | 94·1 |

B—Sarcomata

| International List No. | | Malays | | | | Chinese | | | | Indians | | | |
|---|---|---|---|---|---|---|---|---|---|---|---|---|---|
| | | Male | | Female | | Male | | Female | | Male | | Female | |
| | | No. | % | No. | % | No. | % | No. | % | No. | % | No. | % |
| 196 | Bone | 15 | 2·9 | 14 | 3·0 | 19 | 1·4 | 17 | 1·3 | 9 | 1·7 | 8 | 1·5 |
| 197 | Soft tissues | 38 | 7·4 | 17 | 3·6 | 44 | 3·4 | 27 | 2·1 | 12 | 2·3 | 10 | 1·8 |
| 200 | Reticulosarsarcoma | 28 | 5·5 | 8 | 1·7 | 24 | 1·8 | 19 | 1·5 | 7 | 1·3 | 2 | 0·4 |
| 201 | Hodgkin's disease | 5 | 1·0 | 2 | 0·4 | 9 | 0·7 | 3 | 0·2 | 8 | 1·5 | 3 | 0·5 |
| | Others | 12 | 2·3 | 15 | 3·2 | 13 | 1·0 | 19 | 1·5 | 5 | 0·9 | 9 | 1·7 |
| | Total | 98 | 19·1 | 56 | 11·9 | 109 | 8·3 | 85 | 6·6 | 41 | 7·7 | 32 | 5·9 |
| | Grand Total | 513 | 100·0 | 473 | 100·1 | 1306 | 100·0 | 1282 | 100·0 | 532 | 100·0 | 544 | 100·0 |

It may be surprising that races living together in the same country should differ so much in their cancers, but their environment is not necessarily exactly the same. Malays, Chinese and Indians all tend to cling to their own customs and mode of life; their religions are different, and even their occupations are likely to be different. The Malays are a rural people, rice growers and fishermen. The Chinese are tin miners and merchants, with some now settling on the land as smallholders and market gardeners. The Indians form the labour force on the large rubber estates, though some are in Government service and a few are shopkeepers.

In general it may be said that the Malays and Chinese have a rather similar incidence of their cancers, but in Indians it is usually different. This similarity between the Malays and Chinese is curious as they each have very different customs and mode of life. Genetically, however, they do have more in common, and this may explain the remarkable similarity in so many of their cancers. A similar pattern of genetic susceptibilities could account for the detailed resemblance, as a genetic susceptibility is only to a particular cancer and not to cancer in general.

But there are so many racial differences that cancer in Malaya cannot be described adequately in general terms, and the various cancers must be described separately.

### *The Site Incidence of Cancer*

### *The Alimentary system*

*Oral cavity.*—These cancers would hardly need to be mentioned if it were not for the high incidence of betel cancers among the Indian labourers. Other oral cancers are rather uncommon, probably because they occur rather late in life and there are few old people in Malaya. Most of them are either of the palate, typically developing in a chronic yaws or gummatous ulcer, or of the gum following chronic sepsis or ill-fitting dentures. Cancer of the lip is rare, and of the tongue not common.

The betel cancers form a separate group with characteristic features which distinguish then from the other oral cancers. They are practically confined to Indians of the labouring class: they are rare in Malays and unknown in Chinese. The patients are middle-aged rather than elderly, and it tends to occur earlier in women. The incidence is also a little higher in women than in men at all ages. The site of the lesion is characteristic, being in the buccal sulcus in 80 per cent of these patients.

These cancers are found only among betel chewers, and only those who include tobacco in the quid. This explains the racial incidence of the disease. The Chinese never chew betel; both Indians and Malays do, but only the Indians include tobacco in the quid as the Malays prefer to smoke their tobacco. In the remote parts of the country a few old Malay women may put tobacco in their betel quid, and one or two of them have developed a cancer.

The habits of the Indian betel chewers are responsible for other features of the disease. Indians of the labouring class retain the same betel quid in their mouth for several hours, chewing it for twenty minutes or so and then storing it in the buccal sulcus, but those of a higher class only chew a quid once and then discard it; hence the social class gradient of the disease. These labourers usually commence to chew betel in adolescence. Girls tend to acquire the habit earlier than boys, and women chew betel for longer periods each day than men, so the sex and age incidence are related to the duration and intensity of the habit. The site of the lesion is determined by the way in which the quid is used, for it typically develops where the quid is in most prolonged contact with the mucosa, and that is in the buccal sulcus where it is retained between spells of chewing. The association is very close, for far more of these cancers occur in the left buccal sulcus than in the right and the quid is customarily chewed on the right side but stored in the left, as Mehta *et al.* (1955) described in India.

*Oesophagus.*—Carcinoma of the oesophagus is unusually common in Chinese men but not in the women, nor in the men or women of other races. It is reported to be very common in China, and in North China it is said to equal gastric carcinoma in frequency. It is certainly not so common as that among the South Chinese of Malaya but even in this biopsy material the relative frequency is 6·5 per cent, and it ranks fifth in their cancers.

The suggestion that the high incidence in Chinese is caused by eating very hot food, and that it is only too hot for the men as they are served first, might find support in Malaya where the Chinese use chopsticks but the Malays and Indians eat with their fingers. Unfortunately there is no evidence that the Chinese do eat hotter food than other people, and they certainly have no such reputation in Malaya. The drinking of strong spirits is more likely to be an aetiological factor, for many Chinese men do consume large quantities of spirits while it is rare for Indians to do so, and forbidden to Malays.

*Stomach.*—Gastric carcinoma is much less prevalent than in western countries, although it is still one of the more important cancers. Even in biopsy material it forms 5 per cent of all cancers, and the real frequency must be nearly the 15 per cent found in necropsy series. It is equally common in Chinese and Indians, and affects more men than women in the proportion of 3 to 1 in all races. The incidence in Malays is unknown as the number of cases is inevitably too small because of their aversion to surgery, yet even this number suffices to prove that it is not the rare disease that Bonne (1937) and Kouwenaar (1950) found it to be in the Malays of Java and Sumatra. Differences in the diet may be the principal reason for gastric cancer being less common, but the association with blood group A, first shown by Aird, Bentall and Fraser (1953) must also be concerned as it is less frequent in the Malayan races than in Europeans. The number of pre-pyloric and fundus cancers seems to be proportionately too small, although the site of origin is too often unknown for this to be proved but, if true, it would support Billington's (1956) contention that cancer of the body is not affected by blood group A. Chronic gastric ulcers are not an important aetiological factor although they are common in Chinese and Indians and certainly occur in Malays, as less than 2 per cent of these cancers develop in an ulcer.

*Intestines.*—Carcinoids seem uncommon in the small intestine, but the number of carcinomata developing from adenomatous polyps in young people is surprisingly large. Sarcoma of the intestine occurs in about the expected number but the number of carcinomata of the colon and rectum is much less than in western countries, and even so accessible a carcinoma as that of the rectum is only half as frequent as in London. There are no racial differences in the incidence, and the ratio of men to women affected is about 3 to 2 in all races. Steiner (1954) has called attention to the surprising contrast between the low incidence of intestinal carcinoma in tropical countries and the high incidence of such possible predisposing conditions as parasitic infestations, chronic dysentery and other intestinal infections. In Malaya, at least, no association can be found between carcinoma and such forms of chronic irritation; it is rare to find a carcinoma developing in a bowel ulcer, although the skin cancers typically develop in chronic ulcers and sinuses.

*Liver.*—Primary carcinoma of the liver is very common in Malaya, so much so that a carcinoma in the liver is as likely to be primary as metastatic. It is only the hepatocellular carcinoma which is so common; carcinoma of the intra- and extra-hepatic bile ducts, and of the gall-bladder, is no more prevalent than usual. As in other countries where hepatocellular carcinoma is common, it is only so in men, and it occurs in early middle age. But the disease in Malaya shows some interesting features which may not be so apparent elsewhere. Thus there is a marked racial difference in the incidence, which is very high in Chinese men but much less raised in Indians, the apparent incidence rates being 6·2 and 2·2 per 100,000, respectively. The incidence in Malays is unknown, but is probably as high as in the Chinese because they have the same form of the disease, and are known to have a similar incidence in Java and Sumatra. Again, there is no association with malnutrition, for the Chinese are the best-fed race and have the highest incidence. Liver parasites are also not concerned, for they are rare in Malaya and not found in these patients. Cirrhosis is the most important aetiological factor in these cancers, and it is only those developing in a cirrhotic liver which are increased when the incidence is high. This is so in Malaya where the high incidence in Chinese is entirely due to an increase in the number of those developing in a cirrhotic liver, for they form 95 per cent of their liver cancers but only 80 per cent of those in Indians. But the higher incidence in Chinese is not primarily due to a greater number of cases of cirrhosis, although it is rather more common in Chinese than in Indians; it is the result of a carcinoma developing in more of them, so that the proportion is raised from 8 per cent in Indians to 27 per cent in Chinese. All forms of cirrhosis in Chinese are not equally liable to malignant change; it is only one form, occurring early in life and post-necrotic in type, which is responsible for most of these cancers. The age distribution of the two diseases shows this distinction, for the greatest number of carcinomata is found a decade earlier than that of the cirrhosis.

The particular form of cirrhosis which is responsible for most of the liver cancers in Malays and Chinese is very rarely found in Indians. Their cancers follow other forms of cirrhosis which occur later in life and are not so often followed by malignancy, so the greatest number of their cancers is found a decade later than the maximum number of their cirrhoses. Similarly, the low incidence of hepatocellular carcinoma in women is associated both with a lower incidence of cirrhosis and a lower incidence of malignancy in their cirrhosis.

*Respiratory system*

Carcinoma of the upper respiratory tract is far more prevalent in the Chinese and Malays than in Indians or other races. The susceptibility of the Chinese to these cancers is well known, but that of the Malays is not; neither is it always realised that it is limited to the nose and nasopharnyx and does not extend to the remainder of the pharynx or larynx.

*Nasopharynx.*—Nasopharyngeal carcinoma is an uncommon form of cancer in most people but it is one of the commonest in the Chinese and Malays. The frequency is exaggerated in these statistics as the diagnosis is always confirmed by biopsy, but it is certainly one of the five most frequent cancers in both sexes.

The incidence of carcinoma in the nose and nasal sinuses in Chinese is twice that in Indians but in the nasopharynx it is seven times, the apparent rates being 9·9 and 1·3 per 100,000. Almost all these nasopharyngeal cancers in the Malays and Chinese are of the same histological type, a poorly differentiated epidermoid carcinoma sometimes given the inappropriate name of lympho-epithelioma, and there is no increase in the number of the other types of carcinoma. The natural history of these cancers has a number of features with important clinical implications. They tend to occur earlier than the other nasopharyngeal carcinomata, and the incidence rises very rapidly in the third and fourth decades so that a quarter of these patients are under the age of 40 and two-thirds are under 50. Less than half of these patients when first seen have local symptoms of the growth, and metastases in the cervical glands are usually the first evidence of the disease to be noticed. These often grow much more rapidly than the primary tumour so that the cervical lymph glands may be grossly enlarged while it is still so small and symptomless as to be very difficult to detect. Latency is a common feature of these cancers. The primary growth may give rise to metastases in the cervical lymph glands and then remain latent for years while the metastases grow steadily, spreading from gland to gland and invading the tissues of the neck, so that cerebral nerve lesions are caused by the metastases rather than by the primary growth.

The aetiology is unknown. It is suspected that these cancers are caused by some external carcinogen encountered early in life but none has yet been identified. It must affect Chinese of all social classes equally, and in all parts of the world, so it is difficult to find one which is so universal and yet limited to the Chinese and one or two allied races. The difficulty is greater if it must also affect a race with so different a mode of life as the Malays. Another curious feature is that this carcinogen should be followed by only one histological type of carcinoma. There must surely be more than an external carcinogen to explain this cancer, and a genetic susceptibility must play a large part in the aetiology.

*Lungs.*—Bronchial carcinoma occurs in all the races of Malaya. More men than women develop these cancers in the proportion of 3 to 1, or rather more in Chinese, but only Chinese men have a high incidence of lung cancer. These racial differences may be related to two aetiological factors. If we accept the conclusions of Armitage and Doll (1957) that cell type is related to the aetiology, tobacco can only be an important aetiological factor in Chinese men. For the percentage of Group I of histological types is 73 per cent in Chinese men, which is almost that found in moderate smokers, but in Malays, Indians and women of all races it is only 69 per cent, as in light or non-smokers. This mainly agrees with the extent of the smoking habit in each race and sex. The actual racial consumption of tobacco is unknown, but smoking is a common habit among Chinese and Malay men, uncommon in Indians, and practically unknown among the women of all races. Malay men do not conform to this theory. They are as addicted to cigarette smoking as Chinese, but they have a low incidence of lung cancer and there is no evidence that smoking plays any part in the aetiology. But the incidence of lung cancer is always much lower in the country than in the town, and the rural life of the Malays may explain this anomaly.

### *The Skin*

Skin cancers are very common in Malaya, although not so common as the relative frequency of 10 per cent in these statistics would suggest as it is exaggerated by the large proportion diagnosed, especially in Malays. The Malayan races have a fully pigmented skin and the effect of this is shown in Table II, which compares their skin cancers with those of the Caucasoids living in Malaya.

TABLE II.—*Incidence of Types of Skin Cancer in Malays and Caucasoids Living in Malaya*

| | Malayan races | | Caucasoids in Malaya | |
|---|---|---|---|---|
| | Number | Percentage | Number | Percentage |
| Basal cell carcinoma . . | 91 | 18·8 | 39 | 61·9 |
| Squamous cell carcinoma . | 309 | 63·7 | 17 | 27·0 |
| Malignant melanoma . . | 85 | 17·5 | 7 | 11·1 |
| Total . . . . | 485 | 100·0 | 63 | 100·0 |

The ratio of basal cell to squamous cell carcinoma is reversed, as in Africa (Higginson, 1951), because most of these cancers are caused by chronic sepsis and are therefore squamous cell carcinomata, and there are no actinic cancers, which are more often basal cell carcinomata.

*Basal cell carcinoma.*—The incidence is low, and little more than that of a malignant melanoma. This is due to the absence of actinic cancers rather than to the scarcity of older people, who are just as few among the Caucasoids although they have many more basal cell carcinomata. The incidence appears to be the same in all races and both sexes. It is curious that the distribution of the lesions remains the same despite the absence of actinic cancers. The squamous cell carcinomata without a preceding lesion occur in the same age group, and most of them are also on the head but each keeps to its own typical area.

*Squamous cell carcinoma.*—Most of the skin cancers are of this type, but only because chronic sepsis is so widespread. It was responsible for three-quarters of these cancers in the present series, and the incidence would be even lower than that of the basal cell carcinomata if they were excluded. They are the result of neglected chronic sepsis so it is mainly a disease of the rural population, who seldom seek medical care if it can be avoided and are constantly exposed to minor trauma. The incidence is always higher in men than in women, although the difference is small in Indians, yet even in women chronic sepsis is the cause of most of these cancers. The aetiology determines both the site and the age incidence and it tends to vary with the race and sex. In Malays, most of these cancers develop in a tropical ulcer so they are usually on the leg and occur between the ages of 40 and 60. In Indians and Chinese most of them develop in chronic sinuses and ulcers, the groin is a common site and they occur before or after 40 according to when the infection was contracted. The cancers that occur without a preceding lesion are only found in older people, nearly always after the age of 60 ; more of the cancers in women are of this type, but in both sexes there is the same predilection for the head, especially the scalp and the pinna of the ear.

*Malignant melanoma.*—This is considerably more common in Malayan races than in Europeans, and it forms nearly 2 per cent of all cancers. The incidence in Indians is more than twice that in Chinese; and it is rather higher in men than in women of all races. The incidence may vary but the distribution of the lesions is the same in all these races, and is typical of the disease in a coloured race and not of a tropical country, for Caucasoids living in Malaya retain the typical distribution of their race. Nearly two-thirds of these malignant melanomata were on the foot, most of them on the sole and especially the heel; the conjunctiva was the next most common site despite its small area. The muco-cutaneous junctions, body, hand, arm and leg are occasional sites without any evident predilection for any of them. But it must be emphasised that this is a different distribution of the lesions, consisting equally of an increase in the number of lesions in some areas and a decrease in others, and not just an increase in the number on the foot. It is just as much a part of this change that lesions rarely occur in other areas where they are often found in white races. Thus a malignant melanoma is very rare on the head or neck in a Malayan, and appears to be completely unknown in the uveal tract.

Trauma and chronic irritation of various kinds may be followed by a malignant melanoma, and the frequency with which they are encountered may well affect the incidence. It may explain the higher incidence in Malaya and other tropical countries, but it cannot explain the site of the lesions.

The predilection for the foot is not just the result of the trauma and irritation sustained by bare feet: it is equally evident in those who wear shoes. In the Negro, also, there is the same predilection for the foot whether bare-footed in Africa or in America and wearing shoes (Steiner, 1954).

It is clear that the frequency of a malignant melanoma in a particular site is not entirely determined by the degree of exposure to trauma or other external stimuli. It must also depend on the susceptibility of the melanocytes to malignant change, and where a malignant melanoma will occur is primarily determined by regional variations in this susceptibility. Such regional variations explain why a malignant melanoma should occur on the conjuctiva but not on the uveal tract, as well as the predilection for the foot.

There must also be regional variations in the susceptibility of a benign melanoma to malignant change. They are found anywhere on the body so that their distribution is quite different from that of the malignant melanoma, although they are presumably the origin of more than half of them. The head and neck is a good example of this regional variation. Many benign melanomata occur in this region and they are often of the active junctional type, but not a single malignant one has yet been seen at this Institute. That the site of a benign melanoma can be such a guide to its future behaviour shows how important melanocyte susceptibility can be, and how much it may vary from one region to another.

The distribution of the lesions in this disease must be the result of such regional variations. It does not change with the environment, and so with exposure to external stimuli, but with the race. It is linked to the colour of the skin but not to the degree of pigmentation, as it changes abruptly from one type to the other in white and coloured races. As the type of distribution is determined by race, there must be a genetic factor concerned in these regional variations in melanocyte susceptibility.

### *The Reproductive System*

The reproductive system of women will be considered first, as it is the site of nearly half their cancers but of less than 10 per cent of those in men.

*Breast.*—Only some 2 per cent of breast cancers are sarcomata, most of them developing in a fibro-adenoma which has been long neglected and may be giant in size. The remainder are carcinomata, and they are an important form of cancer in Malaya although little more than half as frequent as the uterine cancers, as is usual when the birth rate is high. The age distribution of breast cancer is the same in all races; the incidence rises steadily to the sixth decade without any second peak such as found in Europe. The incidence is highest in Chinese, and probably almost as high in Malays, but appreciably lower in Indian women. The higher incidence in Chinese women may be related to their greater number of unmarried and childless women, but more to the small number who practice breast feeding.

Carcinoma of the breast in Malaya is curiously uniform in histological type. It is characterised by a very scanty formation of new fibrous tissue. The desmoplastic reaction is so slight that these carcinomata do not become fixed until they invade the skin or the fascia over the pectoralis major. This late fixation may easily mislead the clinician as there may be wide metastasis although the tumour is still mobile.

These carcinomata are always clearly demarcated from the surrounding tissue. On section, the edge is so well defined that the tumour is readily protruded from the surrounding tissue, and may even appear as if it could be shelled out, so that the surgeon has believed it to be encapsulated. There is never any fibrous tissue radiating from these growths, and there was not one typical scirrhous carcinoma among the 300 breast carcinomata in this series. Nearly all these carcinomata originate in the ducts. They may appear somewhat scirrhous when they first invade the surrounding fibrous tissue, but the cells tend to concentrate and not to extend diffusely into the tissues so that the fibrous tissue is destroyed rather than infiltrated. In this way a solid mass of growth with a rather scanty stroma is soon formed. Even microscopically, the edge of the growth may be surprisingly regular with no extensions beyond the advancing line of cells. There is thus no compression of the cells, except in some of the mucoid adenocarcinomata, so they are always large with a big vesicular nucleus.

*Uterus.*—More than a quarter of all the cancers in Malayan women originate in the uterus. Nearly 90 per cent of them are carcinomata, 9 per cent are choriocarcinomata and some 2 per cent are sarcomata, most of them leiomyosarcomata.

*Cervix uteri.*—This is the site of 88 per cent of the uterine carcinomata, the ratio of carcinoma of the cervix to that of the corpus being about 7·5 to 1. The incidence is very high both in Indian and in Chinese women but low in Malay women. Even when all allowance is made for the small number coming to hospital, it is still less than half that in Indians or Chinese. So they are one of the rare instances of a race with a high birth rate and a greater frequency of breast than of cervical carcinomata. The operation of such aetiological factors as the age at first coitus, which is practically the same as the age at marriage in Malaya, and fertility would account for the incidence being rather higher in Indian than in Chinese women, as the Indians marry much younger and have rather more children. But it will not explain why the incidence in Malay women is so much lower than in the other races. It is common for both Malay and Indian girls to marry about the age of puberty, and more than half of them are married before 20. The birth rate is high in all races but highest in Malays, and a third of the Malay women have more than four children compared to a quarter of the Indian.

So another possible aetiological factor must be considered and that is circumcision. The Malays are a circumcised race, and the Chinese, as well as the great majority of the Indians, are not, and it is sometimes claimed that the incidence of cervical carcinoma is low in circumcised races. This is true of the Jews but not of all circumcised races; it is reported not to be true of the Kikuyu, and Nath and Grewal (1935, 1937) did not find much difference between the incidence in the circumcised Muslims and uncircumcised Hindus in India. The number of Indian Muslims in Malaya is small, but there is certainly no evidence that the incidence of cervical carcinoma is low in them as it is in Malay women.

But the low incidence of uterine cancers in Malay women does not resemble that claimed to result from circumcision. It is only carcinoma of the cervix which is said to be less frequent in circumcised races but all uterine carcinomata are equally reduced in Malay women, so that the ratio of carcinoma of the corpus uteri to that of the cervix is similar to that in Chinese or Indians. Thus the ratio in Malay women is 1 to 9, which is very different from the ratio of 1 to 0·3 or 0·68 in Jewesses (Casper, 1955). The low incidence of uterine cancers in Malay women cannot be ascribed to circumcision, and the reason for it is still unknown.

*Corpus uteri.*—The incidence of endometrial carcinoma is low, as might be expected with almost universal marriage and a high birth rate. It is rather higher in Chinese than in Indian women, which is consistent with the differences in marriage customs and fertility mentioned above, but very much lower in Malay women, although maintaining the same proportion to the cervical carcinomata as the other races. It is not likely that there are any more missed diagnoses than in cervical carcinomata, but it is not always realised how much the age structure of the population can affect the numbers of these two cancers. Nearly half of these patients are in the sixth decade, which has only half as many people in it as the fifth decade, the age group with the largest number of cervical carcinomata.

The incidence of sarcoma in all these organs never seems to vary as that of carcinoma does. Sarcoma of the uterus occurs equally in all the Malayan races, and in about the same numbers as elsewhere.

*Choriocarcinoma.*—This occurs much more often in Malaya than in western countries, and in about the same numbers as endometrial carcinoma. As it is a complication of pregnancy it might be expected to occur more often where some 85 per cent of the women have had one or more pregnancies by the age of 30, but this cannot explain all that do occur. The number actually seen at this Institute is a third more than would be expected from the number of live births, and yet it can only be a small fraction of the total. The large number of choriocarcinomata is due to an increase in the number following abortions and hydatidiform moles, so that the proportion following these conditions is raised from the usual 75 per cent to 90 per cent. There is no evidence that more hydatidiform moles become malignant, they are merely more numerous. Abortions are certainly also very numerous, although the number of choriocarcinomata following hydatidiform moles or abortions remains in the ratio of 2 to 1. The high birth rate is responsible for so few of these neoplasms that the proportion following a normal pregnancy is reduced from 22·5 to 6 per cent.

There seems to be a considerable racial difference in the incidence of this disease, as in Chinese it is three times that in Indians, and probably about the same in Malays. This, also, seems to be the result of a similar difference in the number of hydatidiform moles and abortions. The age distribution of choriocarcinoma in Malaya is curious as it follows a markedly bi-modal curve with peaks at 25 and 40 years so that three-quarters of these patients are within five years of those ages.

*Penis.*—In Malaya, carcinoma of the penis is the only important cancer of the male reproductive system. The mixed population well illustrates the role of circumcision and chronic sepsis in the aetiology. The Chinese and the great majority of Indians are uncircumcised but the Malays are Muslims and therefore circumcised. The incidence of carcinoma of the penis is high in Chinese and higher in Indians, but extremely low in Malays. The great difference between the incidence of this cancer in Chinese and Malays, who nearly always have a similar incidence of any cancer, shows how powerful a protection against this disease circumcision can be.

Circumcision is a powerful protection against this cancer but the incidence among the uncircumcised is comparatively low unless some other aetiological factor is present. It is chronic sepsis which is responsible for the incidence being so high in Chinese and Indians, and it is the action of the prepuce in maintaining these infections which makes its presence so important. At least 90 per cent of these patients have had some form of chronic suppuration for many years so that in the absence of sepsis the incidence of carcinoma becomes quite low. It is rarely seen among those Chinese and Indians who have a proper standard of personal hygiene, and will seek adequate treatment for disease.

### *The Nervous System*

*Retinoblastoma.*—This is thought to be more common in eastern countries and is certainly not rare in Malaya, as it forms nearly 1 per cent of all cancers diagnosed here. Of course there will be more if the number of infants in the population is unusually large, as it is in Malaya and many other eastern countries, but they cannot all be explained in this way. If we accept the proportion of 1 in every 34,000 live births which is quoted by Willis (1953), there would be 15 and 4·75 cases among the Chinese and Indians respectively during the five-year period from 1950 to 1954; the actual number seen at this Institute was 5 and 13, and this can only be a fraction of the true number.

About three-quarters of these retinoblastomata are of the undifferentiated type without any degree of rosette formation, and only a quarter are of the more differentiated type with at least poorly formed rosettes. The patients are rarely seen until late in the disease when the difference between these two types in the rate of growth and invasion was clearly seen. In the undifferentiated type the optic nerve was always invaded, and there was extension through the sclera in three-quarters of them, with gross invasion of the orbit in two-thirds. Growth seems to be particularly rapid in the orbit but there is little further local invasion as it fungates outwards when the cavity is filled, although further extension by metastasis through the lymphatics may then occur. In the differentiated type local invasion is much slower; there was invasion of the optic nerve in less than half, and the sclera was invaded in only a third, in none of which was the orbit filled with growth.

### *The Sarcomata*

Sarcomata are often said to be more common in tropical countries. This belief largely arose in the past from errors in diagnosis, but there will be more in proportion to carcinomata in any youthful population because the ratio is higher in the younger age groups. In Malaya they form nearly 7 per cent of all cancers diagnosed by biopsy, although this frequency is probably too high as so many of them are superficial or seriously disabling, but there is no evidence that they are any more frequent than they would be in any other comparable population.

A particular sarcoma may be more or less prevalent than elsewhere, but this is not nearly so common as it is among the carcinomata and they seem to vary much less in their incidence. In Malaya, Hodgkin's disease is the only important instance of such a change in the prevalence of a sarcoma. It is a rare disease among the Chinese and Malays, and certainly much less prevalent than usual. The Indians provide a convenient standard for comparison as it is the same in them as elsewhere. But Hodgkin's disease is only half as frequent in the Chinese and Malays, and the apparent incidence is less than a quarter of that in Indians. The difference can also be shown by comparing the incidence of Hodgkin's disease with that of other sarcomata of lymphoid tissue in each race. In Indians the number of cases of Hodgkin's disease is about the same as the number of reticulosarcomata, but in Chinese and Malays it is less than a quarter of the number. There is no other sarcoma with such a definite difference in its incidence. The primary reticulosarcomata of the soft tissues seem to be more numerous than usual, but it was too often impossible to proved that they were not metastatic and only the prolonged course of the disease in a considerable number of these patients supports the belief. Osteoclastoma appears to occur more frequently in the Chinese, and more than the expected number prove to be malignant by metastasising but the proportion cannot be properly estimated because the metastases may not appear until many years later. Adamantinoma is not a sarcoma, but it is another tumour of bone which appears to be more frequent than usual in Malaya. There is no association with malnutrition or rickets, and it is possible that more are seen merely because it eventually compels the patient to find relief.

### SUMMARY

This survey of the geographical pathology of cancer in Malaya is based on a study of 4650 consecutive cancers diagnosed microscopically by the writer.

More than three-quarters of the cancers occur before the age of 60. This is mainly due to the youth of the population, but there is also a real increase of cancer among the middle-aged. The reasons for this are discussed, and the cancers responsible for the increase are divided into two groups: those which occur earlier because the carcinogen is encountered earlier, and those which are only more prevalent as a result of Malayan conditions.

The site incidence shown by the distribution of these cancers by anatomical site, race and sex is discussed. The population is composed of such different races that they also differ in their cancers. Example of such racial differences are the low incidence of uterine cancers in Malay women, and of Hodgkin's disease in Malays and Chinese; the high incidence of nasopharyngeal cancers in Malays and Chinese, of cancer of the oesophagus and liver in Chinese, and of oral cancers in Indians. So the site distribution of cancer is racial rather than Malayan and cannot be described for the country as a whole. These differences are so numerous that all the more important cancers have had to be considered separately. They are described as they occur in each race, any differences noted, and the variations in the incidence discussed in relation to the aetiology.

### REFERENCES

AIRD, I., BENTALL, H. H. AND FRASER, J. A.—(1953) *Brit. med. J.* **1**, 799.
ARMITAGE, P. and DOLL, R.—(1957) *Brit. J. Cancer*, **11**, 161.
BILLINGTON, B. P.—(1956) *Lancet*, ii, 859.
BONNE, C.—(1937) *Amer. J. Cancer*, **30**, 435.
CASPER, J.—(1955) *Schweiz. Z. Path.*, **18**, 764.
HIGGINSON, J.—(1951) *Cancer*, **4**, 1224.
KOUWENAAR, W.—(1950) ' Symposium on Geographical Pathology and Demography of Cancer '. Oxford, p. 64.
MEHTA, F. S., SANGANA, M. K., BARRETT, M. A. AND DOCTOR, R.—(1955) *J. Amer. dent. Ass.*, **50**, 531.
NATH, V. AND GREWAL, K. S.—(1935) *Indian J. med. Res.*, **23**, 149.—(1937) *Ibid.*, **24**, 633.
STEINER, P. E.—(1954) ' Cancer : Race and Geography '. Baltimore (The Williams & Wilkinson Co.), p. 85.
WILLIS, R. A.—(1953) ' Pathology of Tumours ', 2nd edn. London (Butterworth & Co. Ltd.).